# MDA-MB-231 Breast Cancer Cells and Their CSC Population Migrate Towards Low Oxygen in a Microfluidic Gradient Device

**DOI:** 10.3390/ijms19103047

**Published:** 2018-10-06

**Authors:** Jelle J. F. Sleeboom, Jaap M. J. den Toonder, Cecilia M. Sahlgren

**Affiliations:** 1 Microsystems, Department of Mechanical Engineering, Eindhoven University of Technology, 5600MB Eindhoven, The Netherlands; J.J.F.Sleeboom@tue.nl (J.J.F.S.); J.M.J.d.Toonder@tue.nl (J.M.J.d.T.); 2 Soft Tissue Engineering & Mechanobiology, Department of Biomedical Engineering, Eindhoven University of Technology, 5600MB Eindhoven, The Netherlands; 3 Institute for Complex Molecular Systems, Eindhoven University of Technology, 5600MB Eindhoven, The Netherlands; 4 Cell Fate Lab, Turku Centre for Biotechnology, Åbo Akademi University, FI-20500 Turku, Finland

**Keywords:** cancer stem cells, tumor microenvironment, metastasis, oxygen gradient, migration, microfluidics

## Abstract

Most cancer deaths are caused by secondary tumors formed through metastasis, yet due to our limited understanding of this process, prevention remains a major challenge. Recently, cancer stem cells (CSCs) have been proposed as the source of metastases, but only little is known about their migratory behavior. Oxygen gradients in the tumor have been linked to directional migration of breast cancer cells. Here, we present a method to study the effect of oxygen gradients on the migratory behavior of breast CSCs using a microfluidic device. Our chip contains a chamber in which an oxygen gradient can be generated between hypoxic (<1%) and ambient (21%) conditions. We tracked the migration of CSCs obtained from MDA-MB-231 breast cancer cells, and found that their migration patterns do not differ from the average MDA-MB-231 population. Surprisingly, we found that the cells migrate towards low oxygen levels, in contrast with an earlier study. We hypothesize that in our device, migration is exclusively due to the pure oxygen gradient, whereas the effects of oxygen in earlier work were obscured by additional cues from the tumor microenvironment (e.g., nutrients and metabolites). These results open new research directions into the role of oxygen in directing cancer and CSC migration.

## 1. Introduction

The majority of cancer-related deaths are not caused by the primary tumor, but by the formation of secondary tumors through metastasis [1]. Yet due to our limited understanding of this complex process, preventing the spread of cancer remains a major challenge. Recently, cancer stem cells (CSCs) have been proposed as the source of metastases [2]. CSCs have the ability to self-renew indefinitely, and generate all cell types within the tumor. Although there is still controversy surrounding the hierarchic nature of several types of cancers, considerable experimental evidence for the CSC model has been found: CSCs have been identified and characterized in many different types of tumors, such as pancreatic, colorectal, liver, brain, and breast [3,4].

A close relation between CSCs and metastasis initiating cells is implied by their shared phenotypic and functional properties, such as self-renewal, multipotency, tumorigenicity, therapeutic resistance, and genomic instability [2]. Additional evidence for this relation was found in early breast metastases, where cancer cells were shown to possess a distinct stem-cell like gene expression profile [5]. Additionally, premetastatic circulating tumor cells (CTCs) from the breast share properties with CSCs, such as their tumorigenic potential and expression of CSC markers such as CD44 and Aldehyde Dehydrogenase 1 (ALDH1) [6]. As such, it is becoming commonly accepted that CSCs, or at least a select subpopulation, are drivers of tumor metastasis. However, direct evidence that CSCs shed from the primary tumor form metastases is lacking, possibly due to the challenges in detection, identification, and tracking of CSCs.

The challenges associated with studying metastasis, and especially its early phases, originate from the complexity of the tumor microenvironment (TME) from which metastasizing cancer cells originate. Several cell-extrinsic cues in the TME can directly affect invasive and migratory behavior, such as the extracellular matrix (ECM), mechanics, biochemical cues, and the presence of other stromal cell types [7,8]. Of these cues, the local oxygen concentration is highly relevant for both CSC maintenance and differentiation.

The hypoxic conditions as present in the tumor core have been shown to increase the amount of CSCs in MDA-MB-231 breast cancer cells in vitro [9]. This is attributed to both hypoxia and tissue stiffness in vitro, via the integrin-linked kinase (ILK) mechanotransducer and regulation by the PI3K/ AKT pathway [10,11,12]. Both this relation and stem cell marker expression in cancer cells have been linked to hypoxia inducible factor (HIF) [13,14]. This factor, which responds to hypoxic conditions, also has distinct effects on the migration of cancer cells. It is known to induce the epithelial mesenchymal transition (EMT) in cancer cells via HIF-1 activation mediated by Notch signaling [15]. Through EMT, cancer cells obtain a more migratory phenotype, leading to a loss of cell–cell adhesions and polarity, and increased motility [3]. There seems to be a link between EMT and CSCs, as elevated CSC marker expression is found in cells that have undergone EMT [16]. Although the necessity of EMT for metastasis is still disputed, the role of HIF and hypoxia in directing cancer cell dissemination is well established [17,18].

Recently, breast cancer cells have been shown to respond to not only hypoxia (reduced oxygen), but to a gradient of oxygen [19]. In this study, MDA-MB-231 breast cancer cells migrated towards higher oxygen levels, which has interesting implications for the mechanisms through which they invade and metastasize. It implies that breast cancer cells tend to migrate away from the tumor hypoxic core to invade, which makes the oxygen level inside the tumor, or oxygen-affected signaling pathways, possible targets for metastasis prevention.

However, not much is known about the migratory behavior of the CSC subpopulation, arguably one of the most important properties of metastasizing cells. If these cells are indeed involved in the formation of metastases, understanding their migratory behavior in the complex TME is of key importance. Therefore, learning more about the CSC migration with respect to different TME cues is essential to understanding their role in metastasis. In this work, we focus on the effect of an oxygen gradient on their direction of migration. Our main aim is to find out whether the O_2_ gradient differentially affects the migration of CSCs as opposed to the average cancer cell population in breast cancer.

For this purpose, we developed a microfluidic chip that can generate an oxygen gradient between hypoxic and ambient conditions, and is capable of maintaining a stable gradient for 24 h. Our device facilitates live observation and single cell tracking inside the gradient. We used the device to measure the migration patterns of normal and CSC enriched populations of MDA-MB-231 breast cancer cells, with and without the oxygen gradient.

Our results indicate that the migration patterns in an oxygen gradient of MDA-MB-231 CSCs do not differ from the average cancer cell population. This indicates that either the migration of CSCs and non-CSCs is alike, or that an oxygen gradient alone is not enough to elicit a different response. Interestingly, we find that in contrast to an earlier study [19], MDA-MB-231 cells tend to migrate towards lower oxygen levels. These results open new directions of research into the role of oxygen in directing cancer and CSC migration.

## 2. Results

### 2.1. Oxygen Gradient Chip

**Figure 1:**
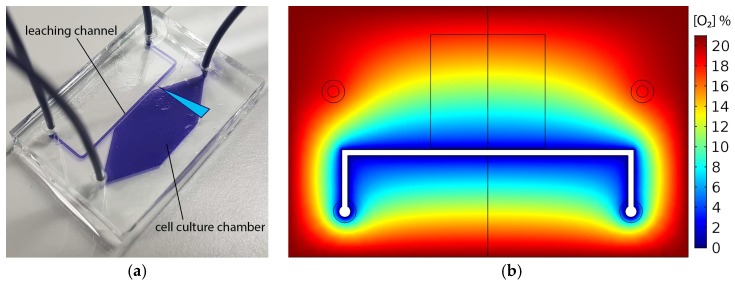
The microfluidic chip with simulated oxygen distribution at the glass cell culture surface: (**a**) Picture of the microfluidic device filled with blue dye. The polydimethylsiloxane (PDMS) is 25 × 30 mm with a height of 2 mm, and bonded to a 25 × 75 mm glass microscope slide. The diffusion barrier on top is 0.5 mm thick, and has the same dimensions as the PDMS. The gradient direction in the chamber is indicated with a blue triangle and (**b**) simulation of the steady-state oxygen distribution in the chip. The chamber outline and chip symmetry line are shown in black.

A polydimethylsiloxane (PDMS) microfluidic device was developed, shown in Figure 1a, containing a chamber in which an oxygen gradient can be generated over a large (10 mm) distance, between hypoxic (<1%) and ambient (21%) conditions. This gradient contains all oxygen levels associated with breast cancer, ranging from median oxygen levels of 1.3% in the tumor, to median levels of 6.8% in healthy breast tissue [20]. The gradient is initiated and maintained by continuous perfusion of a microchannel, adjacent to the chamber, with an oxygen scavenging sodium sulfite (Na_2_SO_3_) solution. Oxygen continuously diffuses from the surrounding air through the PDMS into the leaching channel, which results in a steady-state gradient over the cell culture chamber. The top part of the chip is covered by a 0.5 mm thick Poly(methyl methacrylate) (PMMA) diffusion barrier, which ensures that the oxygen diffuses in-plane. A 3D finite element simulation in COMSOL was used to optimize the chip dimensions. The final design was able to generate an almost linear oxygen gradient over the entire cell culture chamber, as shown in Figure 1b.

**Figure 2:**
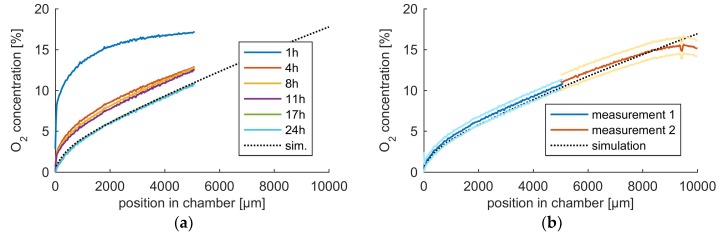
Measured oxygen gradient inside the microfluidic chip: (**a**) The measured oxygen concentration in the chamber at several time points compared to the simulation data and (**b**) the average oxygen concentration across the entire chamber, based on 10 measurements done within 1 h, performed in two parts. The brighter lines indicate the standard deviation of these measurements.

To validate the oxygen profile, oxygen concentration measurements were performed using an oxygen sensitive fluorescent dye, Tris(2,2′-bipyridyl)dichlororuthenium(II) hexahydrate (RTDP). Measurements over time show that the O_2_ gradient shape develops within approximately 4 h, and the predicted concentrations are reached after 17 h, shown in Figure 2a. The concentration profile is in good agreement with the COMSOL simulation, with only small deviations from the computed gradient. The steady-state O_2_ gradient across the entire chamber, measured in two parts after 24 h, showed similar agreement, as shown in Figure 2b. Only at the edge of the chamber, where oxygen levels are highest, a significant deviation from the simulation is found. This deviation is most probably caused by fabrication inaccuracies near the chip edge.

Taken together, the data show that our microfluidic chip is able to generate a stable oxygen gradient over a large distance, in good agreement with the predicted values. The stable gradient between 17 and 24 h enables the tracking of cell migration in constant conditions, making the device well suited for our migration study.

### 2.2. Cancer Stem Cell Migration in an Oxygen Gradient

Using the developed microfluidic oxygen gradient chip, migration experiments were performed with both MDA-MB-231 breast cancer cells, and CSC enriched populations from the same source. The enrichment of CSCs was confirmed experimentally by measuring ALDH activity, shown in Appendix A. In order to investigate their migratory behavior, four conditions were tested: MDA-MB-231 cancer cells with and without gradient and CSC enriched cells with and without gradient. All conditions were tested in three independent experiments, and cell migration was tracked between 17.5 and 24 h after the cells had adhered.

**Figure 3:**
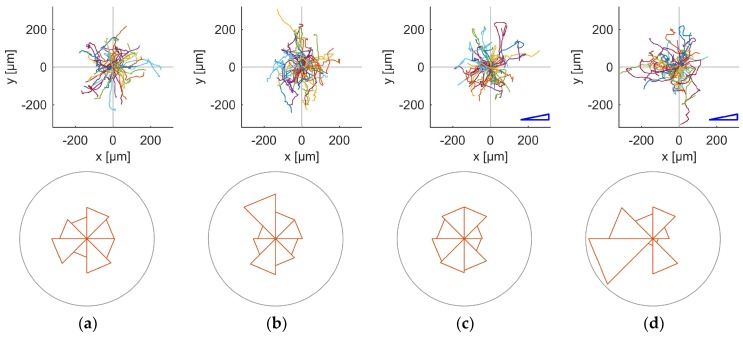
Representative migration tracks and weighted angular histograms of the final cell positions for the four different conditions. A blue triangle shows the direction of the oxygen gradient in the two gradient conditions. The starting points of the migration tracks of each cell are offset to (0,0). Each graph contains the data of all 50 tracked cells in a single experiment: (**a**) Normal MDA-MB-231 cells without gradient; (**b**) CSC enriched cells without gradient; (**c**) normal MDA-MB-231 cells in oxygen gradient; and (**d**) CSC enriched cells in oxygen gradient.

Typical migration tracks are shown in Figure 3, paired with the weighted angular histograms of the final cell positions. In the conditions without oxygen gradient, mostly random migration is observed in the tracking graphs, as visible in Figure 3a,b. At first sight, this is also the case for the normal MDA-MB-231 cells in an oxygen gradient, seen in Figure 3c, but the CSC enriched cells clearly show some deviation towards lower oxygen concentration, as seen in Figure 3d. The corresponding weighted angular histogram also shows this trend. In the weighted angular histogram of CSC without gradient condition, we also see a deviation towards the positive *y*-direction, which is mostly caused by one cell that migrated relatively far in this direction.

In order to understand the migration patterns better, we further evaluate the average forward migration index (FMI), averaged over three independent experiments per condition. This index is a measure for how much of the cell migration is actually used to migrate in a certain direction, and is therefore a better quantitative descriptor of directional migration than the migration tracks and weighted angular histograms.

**Figure 4:**
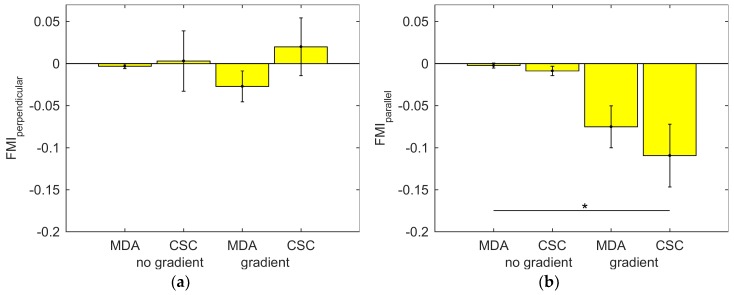
The average forward migration index for all four conditions, defined as the displacement of a cell in the indicated direction divided by the total path length: (**a**) The forward migration index (FMI) perpendicular to the oxygen gradient and (**b**) the FMI parallel to the oxygen gradient. * Statistically significant at *p* < 0.05 using a nonparametric Kruskal–Wallis test.

As a control, we first evaluate the FMI_perpendicular_ as shown in Figure 4a, which is close to 0 for all conditions. This indicates that there is no preferred migration direction perpendicular to the gradient. When we analyze FMI_parallel_ in Figure 4b, both regular cells and cancer stems cells appear to migrate towards lower oxygen levels. However, only the FMI_parallel_ of CSCs in an O_2_ gradient shows a statistically significant difference compared to MDA-MB-231 cells without gradient, and no significant difference is found between MDA-MB-231 cells and CSCs. No significant differences were found in the migration velocities and center of mass displacements, found in Appendix B.

**Figure 5:**
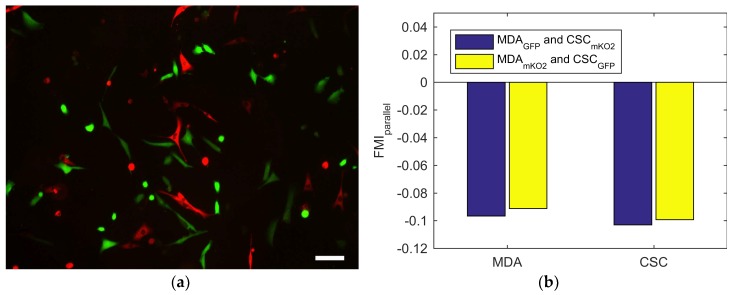
Fluorescent image and the average FMI for mixed populations of cancer stem cell (CSC) enriched and nonenriched MDA-MB-231 cells: (**a**) Representative fluorescent image of a mixed population of CSC enriched MDA-mKO2 cells, and MDA-GFP bulk cells. The scale bar is 100 μm. (**b**) The FMI parallel to the oxygen gradient for one experiment (blue) with MDA-GFP bulk cells and CSC enriched MDA-mKO2 cells (CSC_mKO2_), and another (yellow) with MDA-mKO2 bulk cells and CSC enriched MDA-GFP cells (CSC_GFP_).

To investigate whether the migratory behavior of CSCs was affected by the lack of interplay with the MDA-MB-231 bulk cell population, two experiments were performed with mixed populations of enriched and nonenriched cells. To identify each population, CSCs and bulk cells were obtained from MDA-MB-231 strains that stably expressed either GFP or mKO2 fluorescent protein. Migration experiments were performed with GFP expressing CSCs and mKO2 expressing MDA-MB-231 cells, and vice versa. The FMI perpendicular and parallel to the gradient of these experiments are shown in Figure 5a,b.

The FMI_parallel_ of the two experiments is very similar, both for CSCs and MDA-MB-231 cells. This indicates that in mixed populations, we find the same result as in the separate experiments: Both MDA-MB-231 bulk cells and their CSCs tend to migrate towards lower oxygen levels, with similar FMI. Similar to the previous data, the FMI_perpendicular_, found in Appendix C, indicates that there is no preferred migration direction perpendicular to the gradient in both experiments.

To investigate whether the migratory behavior was affected by the local oxygen concentration, we extracted the FMI_parallel_ and migration velocity at the single cell level. In these data, found in Appendix D, we did not find a clear influence of local oxygen concentration on migratory behavior.

Taken together, the data indicate that an oxygen gradient does not differentially affect the migration of CSCs as opposed to the average MDA-MB-231 cancer cell population, yet it provides us with a surprising result: The direction of migration is opposite to the direction reported in a previous study [19].

## 3. Discussion

In this study, we aimed to investigate whether CSCs migrate differently in an oxygen gradient than the average cancer cell. Our results with separate CSC enriched or bulk MDA-MB-231 cells indicate that this is not the case for MDA-MB-231 breast cancer cells. This also appears to be true for mixed populations of CSC enriched or bulk MDA-MB-231 cells, yet a more detailed study into the behavior of mixed populations is required to fully confirm this. Taken together, this implies that in an oxygen gradient, MDA-MB-231 CSCs do not have a preferred oxygen niche that they migrate towards. It does not disprove the possible existence of a CSC niche in vivo, where, for example, hypoxia driven CSC maintenance and differentiation can still lead to the development of such a niche [11]. However, our data indicate that an oxygen gradient is not a major player in differentially directing MDA-MB-231 cells and their CSCs. This suggests that targeting oxygen gradient related factors, such as HIF, most likely will not lead to selective changes in migration of CSCs, at least not directly via oxygen dependent pathways. Additionally, it might partially explain why metastasis is such an inefficient process [21]: If both CSCs and non-CSCs end up in the circulation as CTCs, only the low number of CSCs that survives the circulation is capable of colonizing a metastatic site.

Since we studied CSC migration in the MDA-MB-231 cell line, verification with other cell lines is still necessary. Importantly, there are differences in the number of CSCs in the average cell population amongst cell lines: MDA-MB-231 cells are known to have a relatively large population of CSCs compared to other breast cancer cell lines, such as the MCF-7 and MDA-MB-468 cell lines [22]. Alternatively, the experiments could be done with specifically isolated cell populations, based on their CSC marker expression, using for example fluorescence activated cell sorting (FACS). Once better knowledge about the underlying mechanisms has been established, the results have to be validated in in vivo settings as well [23].

Our most surprising finding is that MDA-MB-231 cells tend to migrate towards lower oxygen levels, which is in contrast to an earlier study [19]. The difference can possibly be explained by their method to generate an oxygen gradient, which is based on limiting oxygen influx by largely encasing the cells in impermeable glass, relying on cell metabolism to locally deplete oxygen. A downside of this method, as already mentioned by the authors, is that nutrients and metabolites are influenced as well. This means that along with the oxygen gradient, several other gradients can arise. In our method, we generate an oxygen gradient using external control, making it possible to assess the impact of this factor alone, with a lower impact of cell metabolism. A similar external oxygen control based study seemingly confirms this: A549 lung cancer cells also migrated towards lower oxygen levels when exposed to an oxygen gradient [24]. In addition, the direction of migration in our system seems to be in agreement with the typical metastasis path of breast cancer, which is via the lymphatic system [25]. Here, oxygen levels are typically lower, possibly attracting cancer cells via the surrounding oxygen gradients.

At this point, it is important to mention that cues from the TME do not act in isolation, but often synergistically or in competition with other cues. For example, the A549 lung cancer cells were found to exhibit a different response to oxygen gradients in a 3D as opposed to a 2D environment [26], indicating interplay between cues from the ECM and the oxygen gradient. Additionally, other stromal cells in the TME, known to be involved in directing cancer cell migration, have been shown to respond to oxygen levels. One such example are fibroblasts, who have been shown to increase the sensitivity of cancer cells to hypoxic stress [27]. To complicate the TME even further, other soluble cues are known to affect cancer cell migration, and might even be more important than oxygen. One of these cues is provided by epidermal growth factor (EGF), which has been shown to induce higher FMI values of 0.25 to 0.35 in in vitro studies [28,29]. These, and more complex interactions are not modeled or measured in our device, and their relative roles have to be studied in more detail in future work. A good starting point in our system would be to explore different medium conditions and surface coatings, to assess the impact of soluble factors and ECM proteins. Additionally, it would be very interesting to explore the engagement of different signaling pathways, such as HIF dependent pathways. Including these, and any of the other TME cues in our device, is certainly possible, but care has to be taken not to complicate things too much: Controlling and varying cues from the TME independently is still essential to increase our understanding of cancer cell migration. As such, our current study provides insight in the role of oxygen and CSCs in the TME and directions for future research.

## 4. Materials and Methods

### 4.1. Chip Fabrication

The chips were made using standard soft lithography methods [30], with some additional steps to control chip height and include the PMMA O_2_ diffusion barrier. A master mold was produced by spin-coating a 150 μm layer of negative photoresist (su-8 3050, Microchem, Westborough, MA, USA) onto a silicon wafer, placing a photomask on top, and exposing the resist to 8 mW/cm^2^ UV-light for 30 s. The photomask containing the channel geometry was obtained from CAD/Art services, Inc., Bandon, OR, USA, and the design is found in Supplementary File “O2_gradient_chip_mask_design.dxf”. In this design, shown in Figure 1a, the leaching channel is 500 μm wide, and separated from the chamber by a 100 μm wide PDMS wall. The 10 × 10 mm large chamber, with in- and outlet regions that transition from 10 to 1 mm over a distance of 8 mm, is positioned at a distance of 4 mm from the chip side-wall. The leaching channel is positioned over 10 mm from the PDMS side-wall on the opposite side. After a post exposure bake, uncured resin was removed using a developer (mr. Dev 600, micro resist technology GmbH, Berlin, Germany) for 15 min. A casting frame, laser-cut out of 2 mm thick PMMA sheet, was glued to the mold using acrylic glue (Acrifix 1S0116, Evonik, Essen, Germany), to define the width and height of the chip. The design files for the casting frame and the corresponding lids are found in Supplementary Files “O2_gradient_chip_casting_frame_PMMA_2mm.dxf” and “O2_gradient_chip_casting_lids_PMMA_2 mm.dxf”.

PDMS (Sylgard 184, Mavom, Alphen aan den Rijn, The Netherlands) base was mixed with curing agent at a 10:1 ratio, cast into the chip molds, and degassed in a vacuum. Lids were then placed on top of the casting frame to ensure a flat top surface, and the PDMS was cured overnight at 65 °C. The chips were removed from the mold, 1.2 mm in- and outlet holes were punched, and the PDMS slab was bonded to a glass microscope slide. Briefly, the PDMS and glass slides were both exposed to 50 W air plasma for 45 s, brought into contact, and cured at 65 °C for 1 h. To attach the PMMA diffusion barrier, 20 G blunt needle tips (TE720050PK, RS, Haarlem, The Netherlands) were inserted into the in- and outlets, a drop of degassed PDMS was placed onto the chip, and the barriers were pressed on the PDMS to glue them to the chip. The chip was completed by curing it overnight in an oven at 65 °C. The design file for the diffusion barrier is found in Supplementary File “O2_gradient_chip_diffusion_barrier_PMMA_0.5mm.dxf”.

### 4.2. Oxygen Diffusion Simulation

The diffusion of oxygen through our microfluidic chip was simulated using the finite element simulation program COMSOL Multiphysics. In the simulation the chip was modeled as a solid block of PDMS, with an estimated oxygen diffusion coefficient of 3∙10^−9^ m^2^/s [31]. The oxygen leaching channel was set to have a fixed oxygen concentration of 0%, assuming that the influx of oxygen into this channel did not affect the local concentration significantly. The PMMA diffusion barrier was modeled including the access ports present in the real chip, with an estimated diffusion coefficient of 3.7 × 10^−12^ m^2^/s [32]. The oxygen diffusion coefficient in cell culture medium in the chamber was estimated to be similar to the coefficient in water of 40 °C: 3.2 × 10^−9^ m^2^/s [33]. The glass chip bottom was modeled by setting the oxygen flux to zero on the PDMS–glass interface. The surrounding air was assumed to have a constant oxygen concentration of 21%. Both the steady-state and time dependent solutions were computed with a physics dependent mesh, as generated by COMSOL.

### 4.3. Oxygen Concentration Measurement

The oxygen levels in the chip were validated using RTDP (544981-1G, Sigma-Aldrich Zwijndrecht, The Netherlands), a red fluorescent dye that is quenched in the presence of oxygen. From fluorescent intensity measurements, the local oxygen concentration can be computed using the Stern–Volmer equation [34]:

(1) $$\frac{I_{0}}{I}=1+K_{q}\left[O_{2}\right]$$

in which $I_{0}$ is the fluorescent intensity at 0% oxygen, I the current intensity, $K_{q}$ the Stern–Volmer quenching constant, and [$O_{2}$] the current oxygen concentration. Both $I_{0}$ and $K_{q}$ were obtained from two measurements at 0% and 21% (ambient) oxygen levels. These calibration measurements were all performed on the exact same location in the chip as the actual measurements, in order to compensate for measurement errors due to spatial variations in light intensity, refraction, and absorption.

For the 21% oxygen calibration measurement, we filled the culture chamber with a 1 mg/mL RTDP solution in water, and imaged the chamber at 2.5× with a Leica DM4000B-M microscope. In total, 10 images were taken with intervals of 5 min. For the 0% oxygen measurement, the same procedure was repeated, but with an RTDP solution supplemented with 50 mg/mL Na_2_SO_3_ (71988, Fluka) to remove all oxygen from the solution. To obtain local $I_{0}$ and $K_{q}$ values, the images were split into 10 pixel wide strips, perpendicular to the future gradient direction. For each of these strips, the average $I_{0}$ was obtained from the 0% oxygen measurement, after which $K_{q}$ was obtained by solving Equation (1) for the 21% oxygen measurement.

The gradient measurements were then performed by again injecting a 1 mg/mL RTDP solution into the chamber, and connecting a 30 mL syringe with a 50 mg/mL Na_2_SO_3_ solution to the leaching channel. Using a syringe pump (Chemyx, Nexus 3000), the leaching solution was alternately injected and withdrawn through the chip at 2 mL/min to ensure maximum oxygen transfer. Images of the chip were obtained for 24 h every 5 min, and split into the same strips as the calibration data. For each of these strips, the local oxygen concentration was computed using Equation (1).

### 4.4. Cell Culture

MDA-MB-231 human breast adenocarcinoma cells (92020424, Sigma-Aldrich, Zwijndrecht, The Netherlands) were used between P40 and P60. They were cultured in T75 flasks, in 13 mL of normal culture medium, containing RPMI 1640 medium (11875093, Thermo Fisher, Carlsbad, CA, USA) supplemented with 10% fetal bovine serum (FBS) (Bovogen, lot 51113, East Keilor, Australia) and 1% Penicillin/Streptomycin (P/S) solution (SCC0503, Sanbio, Mountain View, CA, USA). Cells were passaged 1:10 when they reached 70 to 80% confluency.

### 4.5. Cancer Stem Cell Enrichment

CSCs were enriched from MDA-MB-231 cells by growing them as spheroids and then selecting for cells growing well without adhering [35]. We experimentally confirmed the CSC phenotype by ALDH activity using the ALDEFLUOR assay as previously described [35], found in Appendix A. The assay, which is based on non-immunological staining of cells with high ALDH expression and FACS detection, was performed according to the manufacturer’s instructions. An almost confluent (80–90%) T75 flask was trypsinized to collect the cells, spun down, and resuspended in 10 mL of CSC enrichment medium, which contained RPMI 1640 medium, 1% P/S, 25 ng/mL EGF (PHG0311, Thermo Fisher), 25 ng/mL basic fibroblast growth factor (bFGF) (F0291-25UG, Sigma-Aldrich, Zwijndrecht, The Netherlands), and B-27 supplement (17504044, Thermo Fisher, Carlsbad, CA, USA). The cells were plated in a 94 mm nontreated petri dish (391–0490, VWR), and kept in culture at 37 °C and 5% CO_2_ for 4 to 5 days. To maximize the selection of CSCs, secondary spheroids were formed by breaking up primary spheroids and replating them in fresh CSC enrichment medium. Briefly, the spheroids were collected from the dish, spun down, and resuspended in 1 mL Trypsin-Versene (LO BE17-161E, Westburg, Leusden, The Netherlands). The spheroids were dispersed by vigorous pipetting, after which the Trypsin was quenched with normal culture medium. The cells were spun down again, resuspended in freshly made CSC enrichment medium, and plated in a new petri dish. Only cells from secondary spheroids were used in the migration experiments.

### 4.6. Migration Experiments

Cell migration experiments were done for four different conditions: normal MDA-MB-231 cells with and without gradient, and CSC enriched MDA-MB-231 cells with and without gradient. In each condition, a standard sterilization and coating protocol was followed: A chip was first sterilized with 100 μL of 70% ethanol for 5 min, after which it was washed three times with 100 μL of phosphate buffered saline (PBS) (LO BE02-017F, Westburg, Leusden, The Netherlands). To coat the chip with 10 μg/cm^2^ fibronectin (FC010, Sigma-Aldrich, Zwijndrecht, The Netherlands), 60 μL of 0.67 mg/mL solution was injected into the chip and incubated at room temperature for 20 min. The chip was then flushed twice with preheated normal cell culture medium, and stored in an incubator until cell seeding.

Cells were seeded in the chip at a density of 6.5 × 10^5^ cells per mL, which roughly compared to 1 × 10^5^ cells per cm^2^. The seeded chip was then incubated for 3 h to ensure adhesion, after which the cell culture medium was carefully refreshed, and the chip was placed on an incubator microscope camera (Lux 10, Cytosmart, Eindhoven, The Netherlands). For the conditions without gradient, image collection was started immediately and continued for 24 h with 10 min intervals. For the gradient conditions, the oxygen leaching equipment was prepared first.

A 50 mg/mL Na_2_SO_3_ leaching solution in water was prepared and aspirated in a 30 mL syringe, which was installed on a syringe pump. The complete pump was placed inside the incubator to avoid generation of temperature gradients on top of the oxygen gradient. The leaching channel of the chip was connected to the syringe and a 50 mL Falcon tube reservoir, and the pump was set to alternately inject and withdraw the leaching solution at 2 mL/min.

Each experimental condition was repeated three times, and migration tracks were obtained manually from 50 cells in each chip, using the MtrackJ plugin in the FIJI image analysis software. The data was further analyzed using MATLAB, and several properties were derived from the data: Weighted angular histograms, the FMI parallel and perpendicular to the gradient, the average migration velocity, and the center of mass displacement. Weighted angular histograms of the final cell positions were based on the angle of the line between the initial and final cell positions. The migration track plots were divided into eight bins, each spanning 45 degrees, and the contribution of each cell was scaled with their distance from (0,0), divided by the average cell distance from (0,0). The FMI parallel and perpendicular to the gradient were defined as the migration distance in the indicated direction divided by total migration path length. The average migration velocity was computed by dividing total migration path length over time. The center of mass displacement was defined as the difference between the final and the initial average cell position of all 50 tracked cells.

### 4.7. Migration Experiments of Mixed Populations

We also performed two migration experiments in the oxygen gradient with mixed populations of MDA-MB-231 bulk cells and CSC enriched MDA-MB-231 cells. For this purpose, we used two MDA-MB-231 strains stably expressing either GFP or mKO2 fluorescent protein, kindly provided to us by Dr. Oscar Stassen. These strains are referred to as MDA-GFP and MDA-mKO2, respectively.

CSC enriched populations were obtained from both strains, and used in the same experiment as described in 4.6., with small differences in the cell seeding and data collection procedures. Briefly, we performed the experiment with mixed populations of either MDA-GFP CSCs and MDA-mKO2 bulk cells, or MDA-mKO2 CSCs and MDA-GFP bulk cells. This to correct for possible effects of the cell line modifications. We seeded the MDA-MB-231 cells and CSCs at a 1:1 ratio with the same density of 6.5 × 10^5^ cells per mL, and performed the experiments with oxygen gradient as described earlier. Afterwards, we imaged the complete chip using a fluorescent microscope (EVOS FL Cell Imaging System, Thermo Fisher, Carlsbad, CA, USA) to manually identify both populations. Tracking data was then obtained for at least 30 cells of each cell strain.

## Figures and Tables

**Figure A1:**
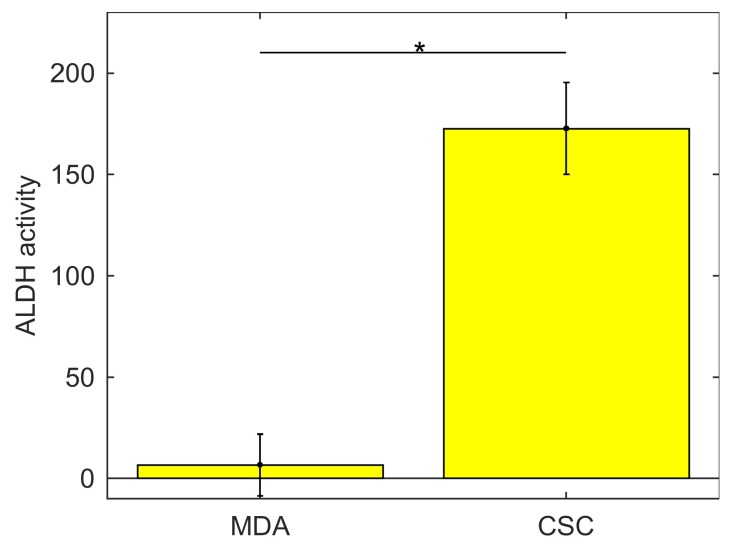
Aldehyde dehydrogenase (ALDH) activity of bulk and CSC enriched MDA-MB-231 cells. Error bars indicate the standard deviation, based on three samples. * Statistically significant at *p* < 0.05 using a nonparametric Kruskal–Wallis test.

**Figure A2:**
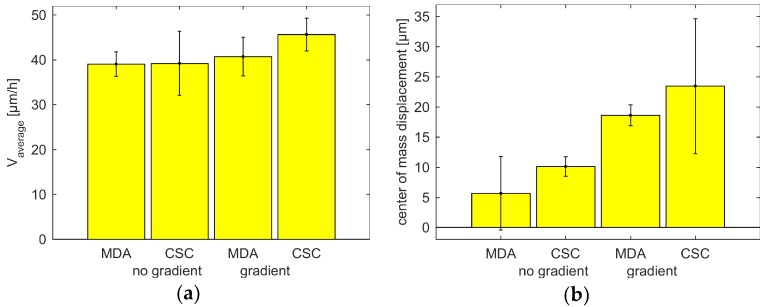
Additional migration properties of MDA-MB-231 cells and their CSC population, averaged over three samples per condition: (**a**) The average migration velocities and (**b**) the center of mass displacements, defined as the total displacement of the average cell position of all 50 tracked cells.

**Figure A3:**
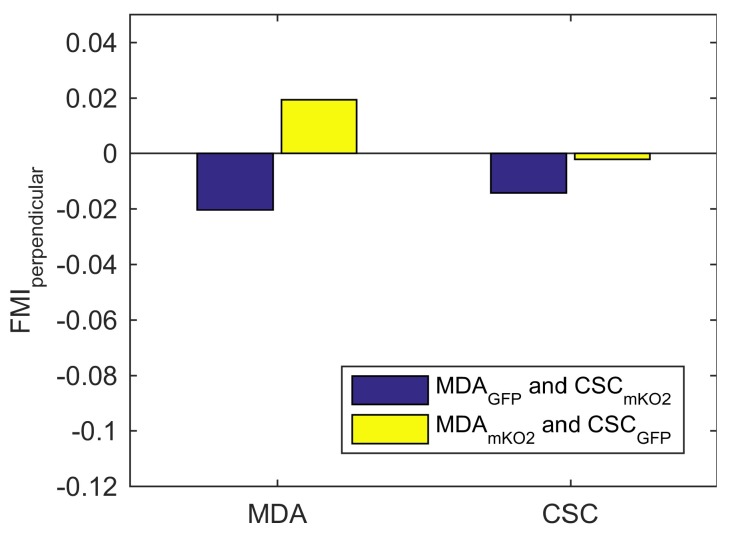
The FMI perpendicular to the gradient, obtained from two experiments with mixed MDA-MB-231 and CSC populations. Data is shown from one experiment (blue) with MDA-GFP bulk cells and CSC enriched MDA-mKO2 cells (CSC_mKO2_) and another (yellow) with MDA-mKO2 bulk cells and CSC enriched MDA-GFP cells (CSC_GFP_).

**Figure A4:**
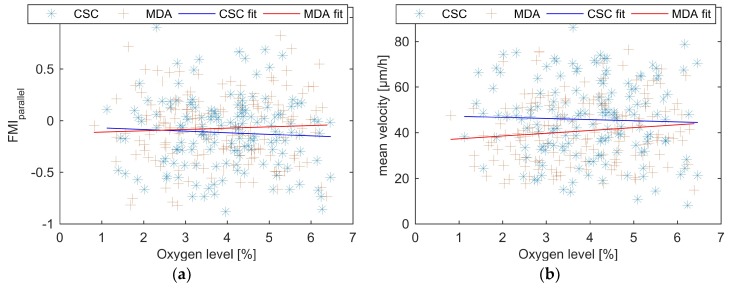
The FMI_parallel_ and average migration velocity in the oxygen gradient, as a function of local oxygen concentration: (**a**) The FMI_parallel_ of all tracked cells and (**b**) the average measured migration velocity of all tracked cells. For both images, the oxygen level experienced by each cell was estimated by taking its average *x*-position, and computing the local oxygen level at that position.

**Figure A5:**
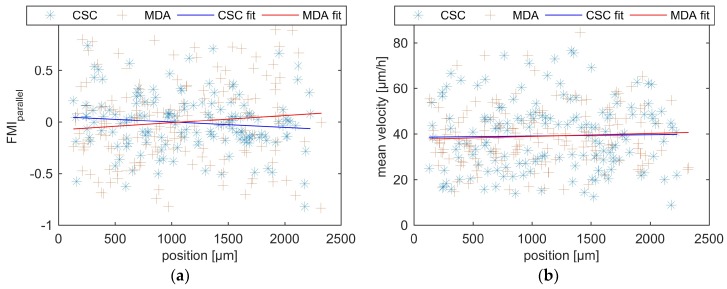
The FMI_parallel_ and average migration velocity without gradient (at environmental oxygen level), as a function of the average x-position relative to the chamber wall: (**a**) The FMI_parallel_ of all tracked cells and (**b**) the average measured migration velocity of all tracked cells.

## Supplementary Materials

Supplementary materials can be found at http://www.mdpi.com/1422-0067/19/10/3047/s1.

## Abbreviations

ALDH1	Aldehyde dehydrogenase 1
bFGF	Basic fibroblast growth factor
CSC	Cancer stem cell
CTC	Circulating tumor cell
ECM	Extracellular matrix
EGF	Epidermal growth factor
EMT	Epithelial–mesenchymal transition
FACS	Fluorescence activated cell sorting
FBS	Fetal bovine serum
FMI	Forward migration index
HIF	Hypoxia inducible factor
ILK	Integrin-linked kinase
PBS	Phosphate buffered saline
PDMS	Polydimethylsiloxane
PMMA	Poly(methyl methacrylate)
RTDP	Tris(2,2′-bipyridyl)dichlororuthenium(II) hexahydrate
P/S	Penicillin/ streptomycin
TME	Tumor microenvironment

## Appendix A

We verified the stem cell phenotype after the CSC enrichment protocol by ALDH activity, as described previously [35]. The ALDH activity in the CSC enriched samples is significantly higher than the baseline activity in MDA-MB-231 cells, see Figure A1. This indicates that the CSC enrichment protocol indeed leads to an increased number of stem cell like cancer cells.

## Appendix B

From the cell migration data, two other quantities are computed: The average migration velocity and the center of mass displacement per condition. The average migration velocity is determined by first averaging the migration velocity per tracked cell, as computed from the displacements and time intervals between images. Next the average is computed for each sample, containing 50 cells. The mean and standard deviation is then computed across all three samples per condition.

The average migration velocity is similar in all conditions, as seen in Figure A2a. No significant differences are found using a nonparametric Kruskal–Wallis test, indicating that the cells in all conditions migrate at the same speed on average.

The center of mass displacement, as seen in Figure A2b, seems to show an increase between conditions, with MDA-MB-231 cells without gradient having the lowest, and CSCs in a gradient the highest value. However, no significant differences are found using a nonparametric Kruskal–Wallis test.

## Appendix C

From the migration experiments with mixed bulk and CSC enriched populations, we also obtained the FMI_perpendicular_. This parameter, shown in Figure A3, is close to 0 for all conditions, indicating that there is no significant bias perpendicular to the gradient.

## Appendix D

In order to check for effects of the local oxygen concentration, the FMI_parallel_ values and average migration velocities were plotted against the average local oxygen concentration experienced by the corresponding cells, shown in Figure A4. As a control, the FMI_parallel_ values and average migration velocities of the no-gradient condition at environmental oxygen level were plotted against the average *x*-position relative to the chamber wall, shown in Figure A5. For both Figures, we pooled the tracking data from the three experiments in each condition. In all scatter plots, linear fits were added to visualize correlation between *x* and *y*-data.

The linear fit of the FMI_parallel_ in Figure A4a seems to indicate that there is a weak but different correlation for the MDA-MB-231 bulk population relative to the CSC population. However, a similar difference between the two populations is found when no gradient is applied, as seen in Figure A5a. Therefore, we conclude that there is no significant effect of the local oxygen level on the FMI_parallel_.

The linear fit of the velocity in Figure A4b also seems to indicate different correlation: CSCs seem to travel slightly faster at low oxygen levels, while MDA-MB-231 bulk cells seem to travel slower. The control without gradient in Figure A5b does not show this difference in correlation, indicating that the effect could be oxygen gradient specific. However, given the relatively weak correlation and the variability found in the FMI data, the significance of this effect is unclear.
